# Development of *Lactococcus lactis* Biosensors for Detection of Diacetyl

**DOI:** 10.3389/fmicb.2020.01032

**Published:** 2020-05-25

**Authors:** Jhonatan A. Hernandez-Valdes, Ana Solopova, Oscar P. Kuipers

**Affiliations:** ^1^Department of Molecular Genetics, Groningen Biomolecular Sciences and Biotechnology Institute, University of Groningen, Groningen, Netherlands; ^2^School of Microbiology, APC Microbiome, University College Cork, Cork, Ireland

**Keywords:** *Lactococcus lactis*, diacetyl, acetaldehyde, biosensor, fluorescence, transcriptional sensor

## Abstract

Some secondary metabolites of fermentative bacteria are desired compounds for the food industry. Examples of these compounds are diacetyl and acetaldehyde, which are produced by species of the lactic acid bacteria (LAB) family. Diacetyl is an aromatic compound, giving the buttery flavor associated with dairy products, and acetaldehyde is the compound responsible for the yogurt flavor and aroma. The quantification of these compounds in food matrices is a laborious task that involves sample preparation and specific analytical methods. The ability of bacteria to naturally sense metabolites has successfully been exploited to develop biosensors that facilitate the identification and quantification of certain metabolites (Mahr and Frunzke, 2016). The presence of a specific metabolite is sensed by the biosensors, and it is subsequently translated into the expression of one or more reporter genes. In this study we aimed to develop fluorescence-based biosensors to detect diacetyl and acetaldehyde. Since the metabolic pathways for production and degradation of these compounds are present in *Lactococcus lactis*, the sensing mechanisms in this bacterium are expected. Thus, we identified diacetyl and acetaldehyde responsive promoters by performing transcriptome analyses in *L. lactis*. The characterization of the biosensors showed their response to the presence of these compounds, and a further analysis of the diacetyl-biosensors (its dynamics and orthogonality) was performed. Moreover, we attempted to produce natural diacetyl from producer strains, namely *L. lactis* subsp. *lactis* biovar diacetylactis, to benchmark the performance of our biosensors. The diacetyl-biosensors responded linearly to the amounts of diacetyl obtained in the bacterial supernatants, i.e., the increases in GFP expression were proportional to the amounts of diacetyl present in the supernatants of *L. lactis* subsp. *lactis* biovar diacetylactis MR3-T7 strain. The biosensors developed in this study may eventually be used to engineer strains or pathways for increased diacetyl and acetaldehyde production, and may facilitate the detection of these metabolites in complex food matrices.

## Introduction

Lactic acid bacteria (LAB) have been used in the production of fermented foods for decades (Song et al., 2017). In the dairy industry, these bacteria utilize lactose as the major carbon source (Neves et al., 2005). Although lactic acid is the major product of lactose fermentation and has a preservative role, LAB metabolism creates other end-products with organoleptic properties like improved flavor or texture, and/or extended shelf-life (Kleerebezemab et al., 2000). Some LAB under different physiological conditions are able to produce acetate, acetaldehyde, acetoin, diacetyl and 2,3-butanediol (Hugenholtz and Starrenburg, 1992; Bintsis, 2018). These minor products are relevant in fermentations. In particular, diacetyl and acetaldehyde are desired flavor compounds in dairy products.

Diacetyl is an aromatic compound known for its buttery aroma and taste. It is found in several dairy products, mainly butter, margarine, sour cream and some cheeses (Rincon-Delgadillo et al., 2012; Clark and Winter, 2015). The amounts of diacetyl reported for dairy products such as margarine, yogurt and Goat milk Jack cheese are 27 ppm (0.31 mM), 200–3000 μg/g (2.1–31.4 mM), and 5.97–13.69 μg/g (0.06–0.14 mM), respectively (Attaie, 2009; Rincon-Delgadillo et al., 2012; Shibamoto, 2014). In addition, diacetyl is naturally present in other products, yet in lower amounts, such as wine and coffee (Clark and Winter, 2015). For instance, the amounts of diacetyl reported for wine and coffee are 0.5–10 mg/L (0.006–0.116 mM) and 2.72 μg/g (0.028 mM), respectively (De Revel et al., 2000; Daglia et al., 2007). Besides its natural appearance in dairy products, diacetyl has a high commercial value and it is manufactured for use as a food additive. Starter distillates (SDs) are also relevant in the formulation of many food products such as cottage cheese and sour cream. The amount of diacetyl in SD ranges from 1.2 to 22,000 μg/g (0.00001–0.22 M). Likewise, acetaldehyde is the major component of the yogurt flavor, which is a mixture of several compounds such as acetone, diacetyl and acetaldehyde (Chaves et al., 2002). Acetaldehyde is present in dairy foods such as yogurt and cheeses in very low amounts, in a range from 73 to 7,000 ng/g (0.002–0.159 M) (Jeong et al., 2015).

A lot of research has focused on the sugar metabolism in *Lactococcus lactis*, the model LAB, which is used as a starter culture for cheese making (Smit et al., 2005). Several engineering strategies using the *L. lactis* MG1363 strain have resulted in the re-routing of carbon metabolism to increase the production of a specific end product such as diacetyl or acetaldehyde (Hugenholtz et al., 2000; Bongers et al., 2005). However, besides the low amounts of the desired compound obtained, and the requirements of strain-engineering methods, their quantification and screening of strains is a laborious work. Biosensors can be utilized as a semi-quantitative tool to facilitate the detection a quantification of these compounds in complex food matrices, which is currently a difficult task that depends on tedious sample preparation methods and analytical methods (Bertels et al., 2012). In essence, genetically encoded biosensors are molecular tools that enable the detection of metabolites in certain matrices in combination with high-throughput screening approaches (Lim et al., 2015).

A biosensor consists of a metabolite-sensing element and a reporting element. In this respect, transcription-based biosensors are metabolite-responsive transcription factors coupled to reporter genes like the genes encoding fluorescent proteins (Fernandez-López et al., 2015; Mahr and Frunzke, 2016). Bacteria are able to respond to many chemical compounds via transcription factors, and the engineering of these sensing systems is achieved by using molecular biology techniques (Zhang and Keasling, 2011). With regard to the utility of the green fluorescent protein (GFP) as a reporting element, a bacterial promoter drives the expression of the GFP, and enables the application of fluorescence activated cell sorting (FACS) as a suitable high-throughput technique. A plethora of biosensors have been developed over the past decades for biotechnological and medical applications (Close et al., 2009; Zhang and Keasling, 2011; Brutesco et al., 2017). One example of transcription-based biosensors is an *E. coli* acetoacetate sensor based on the GFP expression driven by the promoter of the *atoSC* genes encoding a two-component system, which is activated by acetoacetate (Gonzales et al., 2016).

In the present study, we employed transcriptome analysis to identify *L. lactis* genes selectively up-regulated in the presence of diacetyl and acetaldehyde. The candidate promoters that regulate these genes were used to construct fluorescence-based biosensors. The functionality of the biosensors to respond to the presence of the compounds of interest was confirmed by GFP expression. A further characterization of the diacetyl-biosensors was performed to understand the range of diacetyl concentrations to observe response and whether cross-induction by other metabolites is present. Furthermore, we aimed to obtain diacetyl from several producer *L. lactis* subsp. *lactis* biovar diacetylactis strains. To this end, we applied the biosensors to correlate the diacetyl concentration in bacterial supernatants with the fluorescence signals from the biosensors. A concentration of up to 0.42 mM diacetyl resulted in a proportional increase in the GFP measurements. Therefore, we suggest that the biosensors obtained in this work can find application in the quantification of extracellular production of diacetyl and acetaldehyde.

## Materials and Methods

### Chemical Compounds

The chemicals diacetyl 97%, acetaldehyde >= 99.5%, acetoin >= 96%, and 2,3-butanediol 98%, used in this study were purchased from Sigma-Aldrich, St. Louis, MO, United States.

### Bacterial Strains and Growth Conditions

The bacterial strains used in this study are listed in Table 1. *L. lactis* cells were routinely grown as standing cultures at 30°C in M17 broth (Difco^TM^ BD, Franklin Lakes, NJ, United States) or in chemically defined medium (CDM) (Goel et al., 2012), supplemented with glucose (GM17) or lactose (LM17; Sigma-Aldrich, St. Louis, MO, United States) at a concentration of 0.5% (w/v). When appropriate, the culture medium was supplemented with 5 μg mL^–1^ erythromycin.

**TABLE 1:** Strains and plasmids used in this study.

Strain	Description	Reference
***L. lactis***		
MG1363	Opp^+^, DtpT^+^, Dpp^+^, Lac^–^, Prt^–^; plasmid-free derivative of *L. lactis* subsp. *cremoris* NCDO712	Gasson, 1983
RR2	Lac^+^, Prt^+^, *L. lactis* subsp. *lactis* biovar diacetylactis	MolGen collection
WW4	Lac^+^, Prt^+^, *L. lactis* subsp. *lactis* biovar diacetylactis	MolGen collection
M18	Lac^+^, Prt^+^, *L. lactis* subsp. *lactis* biovar diacetylactis	Kuhl et al., 1979
CNRZ190	Lac^+^, Prt^+^, *L. lactis* subsp. *lactis* biovar diacetylactis	Obis et al., 2001
1816	Lac^+^, Prt^+^, *L. lactis* subsp. *lactis* biovar diacetylactis	Obis et al., 2001
CRL264	Lac^+^, Prt^+^, *L. lactis* subsp. *lactis* biovar diacetylactis	Sesma et al., 1990
C17	Lac^+^, Prt^+^, *L. lactis* subsp. *lactis* biovar diacetylactis	Hugenholtz and Starrenburg, 1992
NCDO176 +	Lac^+^, Prt^+^, Cit^+^ *L. lactis* subsp. *lactis* biovar diacetylactis	Bandell et al., 1998
NCDO176-	Lac^+^, Prt^+^, Cit^–^ *L. lactis* subsp. *lactis* biovar diacetylactis	Bandell et al., 1998
IPLA838	Lac^+^, Prt^+^, *L. lactis* subsp. *lactis* biovar diacetylactis	Singh et al., 2003
MR3-T7	Lac^+^, Prt^+^, *L. lactis* subsp. *lactis* biovar diacetylactis	Monnet et al., 2000
P*usp45*-gfp	Ery^r^, MG1363 derivative, *llmg_pseudo10*:P*_*usp*__45_-sfgfp(Bs)*	Overkamp et al., 2013
P*dar*-gfp	Ery^r^, MG1363 derivative, *llmg_pseudo10*:P*_*dar*_-sfgfp(Bs)*	This study
P*plpA-gfp*	Ery^r^, MG1363 derivative, *llmg_pseudo10*:P*_*plpA*_-sfgfp(Bs)*	This study
P*fbaA-gfp*	Ery^r^, MG1363 derivative, *llmg_pseudo10*:P*_*fbaA*_-sfgfp(Bs)*	This study
P*cbiO1-gfp*	Ery^r^, MG1363 derivative, *llmg_pseudo10*:P*_*cbiO*__1_-sfgfp(Bs)*	This study
P*cbiO2-gfp*	Ery^r^, MG1363 derivative, *llmg_pseudo10*:P*_*cbiO*__2_-sfgfp(Bs)*	This study
P*rib-gfp*	Ery^r^, MG1363 derivative, *llmg_pseudo10*:P*_*rib*_-sfgfp(Bs)*	This study
P*0295-gfp*	Ery^r^, MG1363 derivative, *llmg_pseudo10*:P*_0295_-sfgfp(Bs)*	This study
P*2369-gfp*	Ery^r^, MG1363 derivative, *llmg_pseudo10*:P*_2369_-sfgfp(Bs)*	This study
P*1771-gfp*	Ery^r^, MG1363 derivative, *llmg_pseudo10*:P*_1771_-sfgfp(Bs)*	This study
P*butAB-gfp*	Ery^r^, MG1363 derivative, *llmg_pseudo10*:P*_*butAB*_-sfgfp(Bs)*	This study
***E. coli***		
DH5α	F^–^ φ80*lacZ*ΔM15 Δ(*lacZYA-argF*)U169 *recA1 endA1 hsdR17*(rK^–^, mK^+^) *phoA supE44* λ^–^ *thi-1 gyrA96 relA1*	Laboratory stock

**Plasmids**	**Description**	**Reference**

pSEUDO-*gfp*	Ery^r^, integration vector, pSEUDO:*sfgfp(Bs)* derivative, carrying the gene coding for the green fluorescent protein (sfGFP)	Pinto et al., 2011

*Escherichia coli* DH5α strain (Life Technologies, Gaithersburg, MD, United States) was used as the host for cloning and it was grown at 37°C in Luria-Bertani broth or Luria-Bertani agar 1.5% (w/v) (Difco^TM^ BD, Franklin Lakes, NJ, United States). When appropriate, the culture medium was supplemented with 250 μg mL^–1^ erythromycin.

To promote diacetyl production, the *L. lactis* subsp. *lactis* biovar diacetylactis strains (Table 1) were grown in skim milk 10% (w/v) or M17 medium, supplemented with citrate 2% (w/v), and catalase 70 U. Bacterial cultures were grown at 200 rpm in a shaker incubator at 30°C.

M17 and LB-agar plates were prepared by adding agar 1.5% (w/v), and glucose (GM17) or lactose (LM17) to M17. When appropriate, the culture medium was supplemented with 250 μg mL^–1^ erythromycin for *E. coli* and with 5 μg mL^–1^ erythromycin for *L. lactis*.

To corroborate the low lactate dehydrogenase activity in the MR3-T7 strain, bacterial cells were plated on LDAH-20-agar plates. The LDAH-20 was prepared as described previously (El Attar et al., 2000). Briefly, LADH medium is a M17- derived medium containing 2.5 g tryptone, 5 g papain digest of soy beans, 2.5 g peptic digest of meat, 5 g meat extract, 2.5 g yeast extract, 0.5 g L-ascorbic acid, 0.25 g MgSO_4_, 0.5 g K_2_HPO_4_, 10 g glucose. All chemical compounds were dissolved in 1 L distilled water, and the pH was adjusted to 7 with HCl. The medium was autoclaved at 115°C for 15 min, after which its temperature was held at 50°C and 10 ml filter-sterilized 2,3,5-triphenyl tetrazolium (TTC; Sigma-Aldrich, St. Louis, MO, United States) solution (10 g L^–1^) was added. LDAH medium was supplemented with 20 g glycerophosphate, resulting in LAHD-20 medium. LDAH-20-agar plates were prepared by adding agar 1.5% (w/v).

For overnight cultures, flow cytometry analysis and plate-reader assays, *L. lactis* cells were grown in CDM with glucose 0.5% (w/v) and collected by centrifugation from exponential growth cultures (optical density of 0.4 at 600 nm) and washed three times with phosphate-buffered saline (PBS) solution (pH 7.2) containing: KH_2_PO_4_ 15.44 μM, NaCl 1.55 mM and Na_2_HPO_4_ 27.09 μM.

### Recombinant DNA Techniques and Oligonucleotides

Procedures for DNA manipulations (gel electrophoresis and transformation) were performed as described by Sambrook et al. (1989). PCRs were performed in an Eppendorf thermal cycler (Eppendorf, Hamburg, Germany) with *L. lactis* chromosomal DNA as template, using Phusion polymerase (Thermo Fisher Scientific, Inc., Waltham, MA, United States). Oligonucleotides (Table 2) were purchased from Biolegio (Nijmegen, Netherlands). Plasmid DNA and PCR products were isolated and cleaned-up with a High-Pure plasmid isolation kit (Roche Applied Science, Mannheim, Germany), according to the protocol of the manufacturer. Colony PCR and subsequent sequencing (Macrogen, Amsterdam, Netherlands) was used to verify the constructs.

**TABLE 2:** Oligonucleotides used in this study.

Name	Sequence (5′–3′)
PdarFw	CTCTTAGCATGCAGTGGCGACAAACAAGATCAGG
PdarRv	CTAATTTCTCGAGATTTTTCTTCTTTCACAATTTCTAGGA GC
PplpAFw	CTCTGCGCATGCTTTGGAATGAGGCTGATGATGAAGG
PplpARv	CTCCCTTTTCTCGAGTTGATTTATTTTTCAAAATCAATTATT CCCCTTTG
PfbaAFw	GGGTCGATCGAATTCGGTCCTCGGGATATG
PfbaARv	GACTTTGCAAGCTTGCATGCCTGCAGGTCG
P2369Fw	ATCCCTCTCGAGTCCTCACCTTTATAGCAAATTCTC
P2369Rv	GGCATGCCGCATGCTAAATAAGATAGGGAGAATACAT
P0295Fw	CTAACTCAGCATGCGATGATTTTTTTGATACC
P0295Rv	ATTTTCCTCGAGCATCGCTCCTTAGTATTGGTCTTG
P1771Fw	AATTGTCTCGAGTTTCACCTCTTGATTATTTG
P1771Rv	CTTTAAGCATGCACTTGATGCAACAAAAGATA
PribFw	AGAAGCATGCCCATAATACTCATGATAGTAT
PribRv	GCGAGCGCATGCCCAAGTGAGCTGATCATTTATT
PcbiO1Fw	CTGCGCATGCTCAGGAACACTTGATAAGGAATAA
PcbiO1Rv	GACGCTCGAGTAATGGTTCCAGTTTCACCCTTCT
PcbiQ2Fw	CGGCGCATGCTCATTATTATAGTAGGCGGGATTT
PcbiQ2Rv	GAGCCTCGAGCTGTCAGACTTACTTCCTTTATCT
PbutABFw	TAATAGGATTTGGATGTTCTGCTCGAGGACAAA
PbutABRv	GAAATAGCATGCAAAAAATTCTTAGCTTTTTATA

### Construction of Biosensor Strains

We used the *L. lactis* MG1363 strain. All constructed strains are described in Table 1. Candidate promoter regions of the selected up-regulated genes (predicted to be located within the first 300 bp of the non-coding region preceding the open reading frame of the candidate up-regulated gene/transcriptional unit) were selected. To construct the vector pSEUDO:*P_*r*_-gfp*, carrying one of the *L. lactis* MG1363 candidate promoters, the promoter region was amplified by PCR using the Pr_Fw and Pr_Rv, using chromosomal DNA as template. The PCR fragment was cleaved with *Pae*I/*Xho*I enzymes and ligated to pSEUDO-gfp (Pinto et al., 2011). The vector pSEUDO:*P_*r*_-gfp* was integrated into the silent *llmg_pseudo10* locus of *L. lactis* MG1363 by a single-crossover integration as described previously (Overkamp et al., 2013). Transformants were selected on M17-agar plates supplemented with sucrose, glucose and erythromycin 5 ug mL^–1^, yielding the *L. lactis P_*r*_-gfp* strains.

### Growth Curves

The *L. lactis* MG1363 strain was grown in the presence of diacetyl or acetaldehyde in GM17 broth. *L. lactis* cells were grown overnight at 30°C in GM17. Overnight cultures were diluted 1:20 in 10 mL of fresh GM17 and grown at 30°C until exponential growth phase (optical density of 0.4 at 600 nm). The chemical compounds were added at the following concentrations: diacetyl at 1, 5, 10, 30 mM, and acetaldehyde at 0.005, 0.02, 0.1, 0.2, 0.7, 1, 2, and 2.5 M. Acetaldehyde is extremely volatile. Therefore, the tubes were closed tightly after the compound was added (see Supplementary Figure 1). Growth curves for diacetyl-treated cultures were obtained by measuring the optical density at 600 nm (OD_600_) every 30 or 60 min. Final cell densities in acetaldehyde-treated cultures were obtained by measuring the optical density at 600 nm of the bacterial cultures after 5.5 h of incubation.

### Sampling and RNA Isolation

Cultures of *L. lactis* were grown and prepared as described above. At exponential growth phase (OD_600_ = 0.4) diacetyl was added at a concentration of 5 mM, and acetaldehyde at a concentration of 0.7 M. After 30 min, the equivalent of 10 OD_600_ units of volume (volume in mL multiplied by OD_600_) was taken. Cells were collected by centrifugation in 50 mL Greiner tubes in an Eppendorf 5810R centrifuge (Eppendorf AG, Hamburg, Germany), during 10 min centrifugation, speed 10,000 rpm, at 4°C. Cells were resuspended in 0.5 mL of TE buffer (10 mM Tris-HCl, 1 mM Na_2_-EDTA, pH 8.0), prepared with diethylpyrocarbonate (DEPC)-treated deionized water. Cells were transferred to a 2 mL screw cap tube, immediately frozen in liquid nitrogen and then kept at −80°C.

For RNA isolation, 0.5 g of glass beads (∼100 μm in diameter), 50 μL of 10% sodium dodecylsulfate (SDS; Sigma-Aldrich, St. Louis, MO, United States), 500 μL of premixed phenol:chloroform:isoamylalcohol (25:24:1) and 175 μL of macaloid suspension were added to the thawed cells in the screw cap tube. Cells were disrupted using 2 cycles of 45 s of bead beating with a 1 min interval on ice. The cell lysate was cleared by centrifugation in an Eppendorf 5417R centrifuge (Eppendorf AG, Hamburg, Germany) during 10 min centrifugation, speed 10000 rpm, at 4°C. Next, the upper phase was extracted with 500 μL of chloroform: isoamylalcohol (24:1). The two phases were resolved by centrifugation (10 min, 10000 rpm, 4°C) and total RNA was isolated from the aqueous phase using the High Pure RNA Isolation Kit (Roche Molecular Biochemicals, Mannheim, Germany), according the manufacturer’s instructions. RNA concentration was determined with a NanoDrop ND-1000 spectrophotometer (NanoDrop Technologies, Wilmington, DE, United States); RNA quality was assessed using an Agilent Bioanalyzer 2100 with RNA 6000 LabChips (Agilent Technologies Netherlands BV, Amstelveen, Netherlands).

### DNA Microarray Procedure

Synthesis of copy DNA (cDNA) was performed using the Superscript III Reverse Transcriptase kit (Invitrogen, Carlsbad, CA, United States). Incorporation of amino allyl-modified dUTPs during cDNA synthesis allowed Cy3/Cy5 labeling with the CyScribe Post labeling Kit (Amersham Biosciences, Piscataway, NJ, United States), according to the supplier’s instructions. All intermediate and final purifications of either labeled or unlabeled cDNA were performed with a NucleoSpin Extract II Kit (Clontech Laboratories, Mountain View, CA, United States), according to manufacturer’s instructions except when purifying unlabeled cDNA, where 80% ethanol was used in a second washing step and 0.1M sodium bicarbonate, pH 9.0, was used as the elution buffer.

Hybridization to the probes spotted on the *L. lactis* MG1363 mixed amplicon and oligonucleotide DNA microarray slides, covering 2308 of the 2435 predicted ORF’s, was done using the Slidehyb 1 hybridization buffer (Ambion Biosystems, Foster City, CA, United States), during 16 h at 45°C. After hybridization, slides were washed for 5 min in 2X SSC (150 mM NaCl and 15 mM trisodium citrate) with 0.5% SDS, 2 times for 5 min in 1X SSC with 0.25% SDS and 5 min in 1X SSC with 0.1% SDS. All washing steps were performed at 30°C with preheated buffers. The washing buffers were removed via centrifugation (Eppendorf 5810R, 2 min, 2000 rpm). The DNA microarray slides were scanned using a GenePix Autoloader 4200AL confocal laser scanner (Molecular Devices Corporation, Sunnyvale, CA, United States). The resulting images were analyzed using the ArrayPro Analyzer 4.5 software (Media Cybernetics, Silver Spring, MD, United States). Signal intensities were quantified for each spot on the DNA microarray slides after subtracting the background intensities, which were determined for each spot by reading the signals in the regions that separated diagonal spots. Signals were initially normalized and scaled via LOWESS using the MicroPrep software (van Hijum et al., 2003), after which a Dimensioning-Noise-Amplitude (D-N-A) scaling was performed.

### Flow Cytometry

*Lactococcus lactis* cultures were grown overnight in CDM as described above, washed three times in PBS and transferred to fresh CDM supplemented with diacetyl (3.5 mM) or acetaldehyde (0.5M). The cultures were incubated at 30°C and samples were taken at beginning of the stationary growth phase. The GFP-signal in all samples was recorded in 10,000 events (cells) and used for downstream analysis (named ungated events in the corresponding figures). GFP-signal measurements were obtained with a FACS Canto flow cytometer (BD Biosciences, San Jose, CA, United States) using a 488 nm argon laser. A threshold for the FSC and SCC parameters was set (200 in both) in the FACS Canto flow cytometer (BD Biosciences, San Jose, CA, United States) to remove all the events that do not correspond to cells. Raw data was collected using the FACSDiva Software 5.0.3 (BD Biosciences). And the FlowJo software was used for data analysis^1^.

To benchmark the performance of the biosensor to detect diacetyl in bacterial supernatants, 700 μL of filtered *L. lactis* subsp. *lactis* biovar diacetylactis supernatant were used for induction, or 700 μL of a diacetyl solution (0.42 mM) as control. 500 μL of fresh CDM were added to grow the biosensor cells. The cultures were incubated at 30°C and samples were taken at beginning of the stationary growth phase. The GFP-signal at single-cell level was recorded in 10,000 ungated events as described above.

### Plate Reader Assays

Cultures of *L. lactis* were grown and prepared as described above. For fluorescence intensity measurements, *L. lactis* cells were diluted 1:20 in CDM. When testing the effect of varying concentrations of diacetyl, acetoin or 2,3-butanediol, CDM was used and supplemented with different compound concentrations (diacetyl 1.2, 1.7, 2.3, 2.9, 3.5, 4.1, 4.6 mM; acetoin 0.11, 0.17, 0.23, 0.28, 0.34, 0.4, 0.45 M; and 2,3-butanediol 0.22, 0.33, 0.44, 0.55, 0.67, 0.78, 0.89 M). The growth and fluorescence signal were recorded in 0.2 mL cultures in 96-well micro-titer plates and monitored by using a micro-titer plate reader VarioSkan (Thermo Fisher Scientific, Inc., Waltham, MA, United States). Growth was recorded with measurements of the optical density at 600 nm (OD_600_) and the GFP-signal was recorded with excitation at 485 nm and emission at 535 nm every 10 min for 24 h. Both signals were corrected for the background value of the corresponding growth medium. The highest GFP-signals in relative fluorescence units (RFUs) were normalized by the corresponding OD_600_ measurements, yielding RFU/OD_600_ values.

### Voges-Proskauer (VP) Test

*Lactococcus. lactis* cells were grown as standing cultures at 30°C in peptone-glucose broth (MR-VP broth) for 28 h. This medium was prepared with pancreatic digest of casein 7.0 g, dipotassium phosphate 5.0 g, and dextrose 5.0 g. The components were dissolved in 1 L deionized water, and pH adjusted to 6.9. The medium was autoclaved at 115°C for 15 min.

The Voges-Proskauer reagents were freshly prepared, Barritt’s reagent A: 5% (w/v) 1-naphtol (Sigma-Aldrich, St. Louis, MO, United States) in absolute ethanol. Barritt’s B: 40% (w/v) KOH in deionized water. The VP test was performed as reported previously (Mcdevitt, 2009). Briefly, 0.6 mL of Barritt’s reagent A were added to 2.5 mL of the bacterial cultures, then 0.2 mL of Barritt’s reagent B were added. The tubes were shaken for 30 s to expose the culture to atmospheric oxygen, and allowed to stand for 30 min. Within 1 h the tubes were compared. A yellowish color indicates VP-negative and a red color indicates VP-positive.

### Quantification of Pyruvate Metabolites

Acetoin and 2-acetolactate concentrations were obtained colorimetrically using the method developed by Westerfeld (1945). The diacetyl concentration was obtained using the method developed by Elliker (1945) and Westerfeld (1945). Acetoin and 2-acetolactate concentrations were determined as follows. 200 μL of each filtered bacterial supernatant were used. 400 μL of NaOH 1 M (for acetoin quantification) or HCl 0.5 M (for 2-acetolactate quantification) were added to the supernatant. Samples were incubated at 44°C for 30 min. 400 μL of deionized water, 1 mL of creatine 0.5% (w/v) and 1 mL of 1-naphtol 5% (w/v) were added. The reactions were incubated at 20°C for 60 min and absorbance was measured at 525 nm using a plate reader. The 2-acetolactate (α-acetolactate) concentration is obtained with the formula HCl treated sample = [decarboxylated α − acetolactate] + [acetoin]. And the results were multiplied by the factor 100/62 (a 62% of 2-acetolactate standard is transformed to acetoin under the assay conditions). A standard curve was measured using acetoin (0.1–2.4 mM). To quantify diacetyl, 680 μL of filtered supernatant were used. Next, 20 μL of 3,3- diamino benzidine tetrahydrochloride 0.5% (w/v) were added. After incubation for 1 min at room temperature, 200 μL of H_2_SO_4_ 3M and 100 μL of water were added. Samples were incubated for 10 min at room temperature, and absorbance was measured at 366 nm using a plate reader. A standard curve was measured using diacetyl (0.01–1 mM).

### Statistics and Reproducibility

Statistical analyses were performed using Prism 6.01 (GraphPad software^2^) and R v3.3.0. All experiments were repeated independently at least three times.

### Bioinformatics

Promoters were identified in the met promoter region were analyzed with PePPER (de Jong et al., 2012). Output Visualization of the gene expression profiles was studied and depicted using R 2.15.1 software packages^3^.

### Accession Numbers

Gene expression data were deposited in the GEO database under accession numbers: GSE147695 and GSE147696.

## Results

### *L. lactis* Growth in the Presence of Diacetyl and Acetaldehyde

**FIGURE 1:**
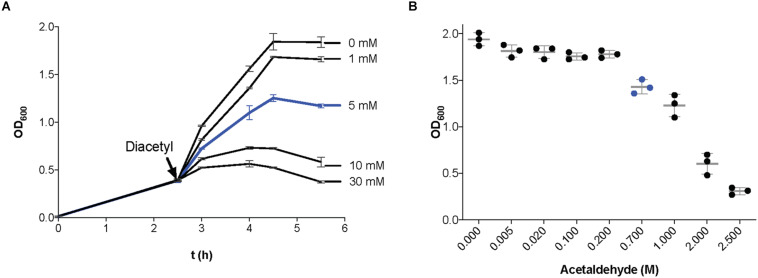
Effect of the presence of diacetyl and acetaldehyde on *Lactococcus lactis* growth. **(A)** Growth curves (optical density at 600 nm; OD_600_) of bacterial cultures in GM17. When the cultures reached an OD_600_ = 0.4 (black arrow), incubation continued in the presence of different concentrations of diacetyl (1–30 mM). A concentration of 5 mM diacetyl (line in blue) was used for transcriptome analysis. **(B)** Final optical density at 600 nm of *L. lactis* cultures incubated in GM17 in the presence of different concentrations of acetaldehyde (0.005–2.5 M). A concentration of 0.7 M acetaldehyde (blue dots) was used for transcriptome analysis. Data are presented as mean ± SD. Error bars represent standard deviation (SD) of the mean values of three independent experiments.

To determine the effect of diacetyl and acetaldehyde on cell growth of our model strain *L. lactis* MG1363, we measured the optical density at 600 nm (OD_600_) of bacterial cultures in the presence of different concentrations of diacetyl and acetaldehyde (see section “Materials and Methods”). Figure 1A shows the *L. lactis* growth curves in the presence of diacetyl. At a concentration of 5 mM (blue line), the bacterial culture is able to grow, but reaches a lower final cell density compared to the untreated culture. In contrast to concentrations above 10 mM, where the growth curves of bacterial cultures show a decline phase and a low final cell density. These results are in agreement with a previous study of the antimicrobial properties of diacetyl, where LAB were unaffected by the presence of diacetyl at a maximum concentration of 4 mM (Jay, 1982). Figure 1B shows the final cell density of *L. lactis* cultures in the presence of acetaldehyde. This compound is extremely volatile (boiling point approximately 21°C; Supplementary Figure 1) (Osborne et al., 2000), and thus, single measurements of the final cell densities of bacterial cultures in the presence of acetaldehyde were measured. At a concentration of 0.7M (blue dots), the bacterial culture is able to grow, but reaches a lower final cell density compared to the untreated culture. In contrast, the bacterial cultures with acetaldehyde at concentrations above 2M show very low final cell densities. Although the antimicrobial properties of acetaldehyde have been reported against *Escherichia coli* (1 mM) (Vandenbergh, 1993), the inhibitory concentration has not been calculated for Gram-positive bacteria.

### Identification of Diacetyl- and Acetaldehyde-Responsive Genes by Transcriptome Analysis

We performed a transcriptome analysis of *L. lactis* cells treated with either diacetyl or acetaldehyde in order to study gene expression changes upon exposure to these compounds. The transcriptome analysis was performed after 30 min exposure to the compounds. Notable differences in global gene expression profiles between treated and untreated samples were observed (see Supplementary Figure 2 and Data File). A differential expression analysis of all the genes is shown in the Supplementary Material. Next, we selected genes up-regulated in the presence of diacetyl or acetaldehyde, and identified the promoters controlling the expression of these genes (Tables 3, 4). The selection of these candidate responsive promoters is primarily based on the genes with a high fold change value, and secondly whether the up-regulated genes belong to single transcriptional units, except for the gene *llmg_025*, which is a single gene but showed a high level of up-regulation (25-fold; see Table 3). With regard to the latter criteria, the selection of promoters controlling transcriptional units composed of more than one gene minimizes type I errors (minimizing false positives) (Junier and Rivoire, 2016), i.e., the selection of one promoter that regulates the expression of a set of up-regulated genes because these genes belong to a single transcriptional unit. Importantly, Table 3 shows two possible promoters in the predicted transcriptional units, i.e., *dar-plpD* and *cbiOQ* (see Supplementary Figure 3A), and both promoters were considered for further analysis.

**TABLE 3:** Candidate responsive promoters to diacetyl.

Promoter	Gene	FOLD up-regulation	adj. *P*-value	Product
P*dar*/ P*plpA*	*dar*	2.2	6E-03	Acetoin(diacetyl)reductase
	*plpD*	2.3	2E-02	D-Methionine-binding lipoprotein
P*rib*	*ribA*	3.3	2E-02	Riboflavin biosynthesis protein
	*ribB*	4.2	5E-03	Riboflavin synthase subunit alpha
	*ribD*	4.9	4E-03	Riboflavin biosynthesis protein
	*ribH*	6.6	4E-03	6,7-Dimethyl-8-ribityllumazine synthase
P*cbiO1*/ P*cbiO2*	*cbiO*	2.4	5E-03	Cobalt ABC transporter ATP-binding protein
	*cbiQ*	2.6	2E-02	Cobalt ABC transporter permease
P*0295*	*llmg_0295*	25.6	4E-04	Hypothetical protein
P*2369*	*llmg_2368*	16.3	2E-04	Hypothetical protein
	*llmg_2369*	9.2	6E-04	Hypothetical protein

*Differentially expressed transcriptional units or single genes upregulated as a result of the presence of diacetyl in the medium. Genes with a differential expression ≥ 2.5 fold with a P ≤ 0.05, or previously reported in the literature to be related to diacetyl were selected.*

**TABLE 4:** Candidate responsive promoters to acetaldehyde.

Promoter	Gene	FOLD up-regulation	adj. *P*-value	Product
P*butAB*	*butA*	2.9	1E-03	Acetoin reductase
	*butB*	2.6	7E-04	2,3-Butanediol dehydrogenase
P*1772*	*llmg_1768*	4.1	2E-04	Hypothetical protein
	*llmg_1769*	7.9	6E-04	Hypothetical protein
	*llmg_1771*	5.5	1E-03	Rhodanese-related sulfurtransferase
	*llmg_1772*	6.9	3E-04	Rhodanese-related sulfurtransferase
	*noxC*	9.3	2E-04	NADH oxidase
P*1639*	*llmg_1639*	10.3	1E-04	ABC transporter ATP-binding protein
	*llmg_1640*	10.3	1E-04	Hypothetical protein
P*mal*	*malE*	9.4	1E-05	Maltose ABC transporter substrate binding protein
	*malF*	17.4	6E-06	Maltose transport system permease malF
	*malG*	13.8	2E-05	Maltose ABC transporter permease malG
P*1729*	*copA*	8.1	6E-05	Copper/potassium-transporting ATPase
	*merP*	3.3	2E-03	Mercuric reductase
	*copR*	3.1	2E-03	Transcriptional regulator

*Differentially expressed transcriptional units or single genes upregulated as a result of the presence of acetaldehyde in the medium. Genes with a differential expression ≥ 2.5 fold with a *P* ≤ 0.05 were selected.*

### Development of Biosensors

**FIGURE 2:**
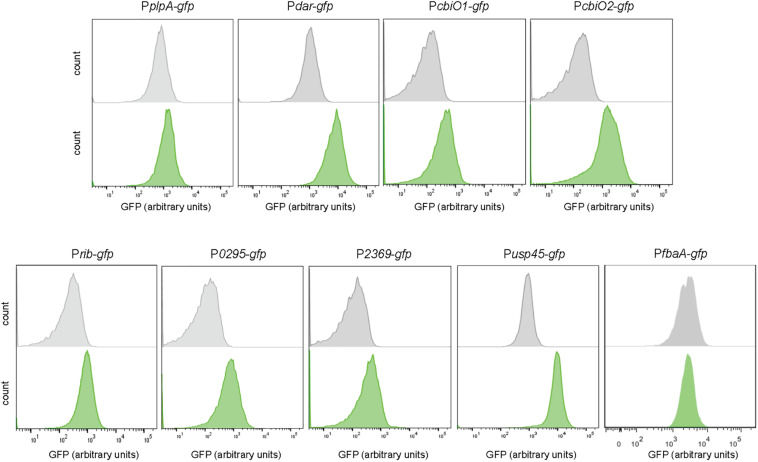
Diacetyl-inducible biosensors. Single-cell fluorescence measurements by flow cytometry, in the absence (gray) and presence of 3.5 mM diacetyl (green). Fluorescence measurements were taken at the beginning of stationary growth phase. 10,000 ungated events for each sample are shown.

The DNA sequences of the candidate promoters were cloned upstream the gene encoding the GFP. Competent cells of *L. lactis* were transformed with the vectors bearing the transcriptional fusions to obtain the fluorescent-based biosensors (Supplementary Figure 3B). Figure 2 shows the response of the constructed biosensors to diacetyl. The P*dar-gfp* strain shows higher fluorescence levels in the presence of diacetyl, in contrast to the P*plpA-gfp* strain, which shows the same fluorescence levels in the treated and untreated sample. The response of these two candidate promoters for the transcriptional unit *dar-plpD* (P*plpA-gfp* and P*dar-gfp*) confirms that only P*dar-gfp* is induced with diacetyl. The response of the selected promoters for the transcriptional unit *cbiOQ* (P*cbiO1-gfp* and P*cbiO2-gfp*) shows induction in both, although P*cbiQ2* is more sensitive to diacetyl as it reaches higher fluorescence intensity in the presence of diacetyl than P*cbiO1-gfp*. Moreover, higher GFP expression levels are also observed in P*rib-gfp*, P*0295-gfp*, and P*2369-gfp* when the strains are exposed to diacetyl.

To validate our results, we aimed to test the response of a constitutive promoter to the presence of diacetyl. The *usp45* promoter has been previously described as a strong promoter in *L. lactis* (Li et al., 2011). A strain bearing a transcriptional fusion with the *usp45* promoter was constructed as described above. In an unexpected way, the P*usp45-gfp* strain responds to diacetyl as well, and a clear separation of treated and untreated cells is observed. However, our transcriptome analysis shows only a 1.02 fold-change value for the *usp45* gene. It is important to mention that microarrays tend to have a low dynamic range, which results in under-representations of fold changes in gene expression (Mutch et al., 2001). For instance, highly expressed genes, such as *usp45*, tend to have little variance, and a small fold change for these genes might be relevant. To validate our observations, we constructed another transcriptional fusion with the promoter P*fbaA*. The *fbaA* gene encodes the fructose-bisphosphate aldolase, a key enzyme in the glycolysis pathway, i.e., it has a housekeeping role in metabolism (Shams et al., 2014). The P*fbaA-gfp* strain does not respond to diacetyl and, just as the non-inducible P*plpA-gfp* strain, is a suitable control to validate our results.

**FIGURE 3:**
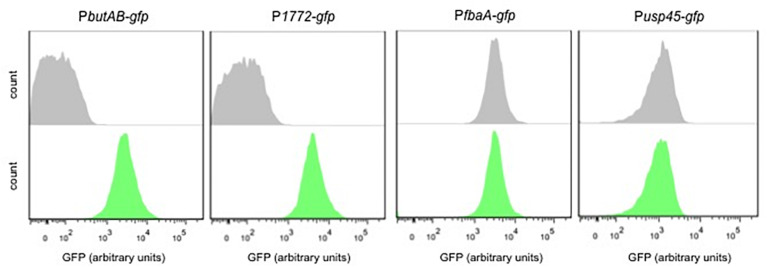
Acetaldehyde-inducible biosensors. Single-cell fluorescence measurements by flow cytometry, in the absence (gray) and presence of 0.5 M acetaldehyde (green). Fluorescence measurements were taken at the beginning of stationary growth phase. 10,000 ungated events for each sample are shown.

Two transcriptional fusions were tested for acetaldehyde. Figure 3 shows that the P*butAB-gfp* and P*1771-gfp* strains respond to acetaldehyde. Moreover, in both strains a clear separation between the treated and untreated samples is observed. To validate these findings, the strains P*usp45-gfp* and P*fbaA-gfp* were also exposed to acetaldehyde. Certainly, both strains P*usp45* and P*fbaA* do not respond to acetaldehyde and are suitable controls.

### Specificity and Sensitivity of Diacetyl Biosensors

**FIGURE 4:**
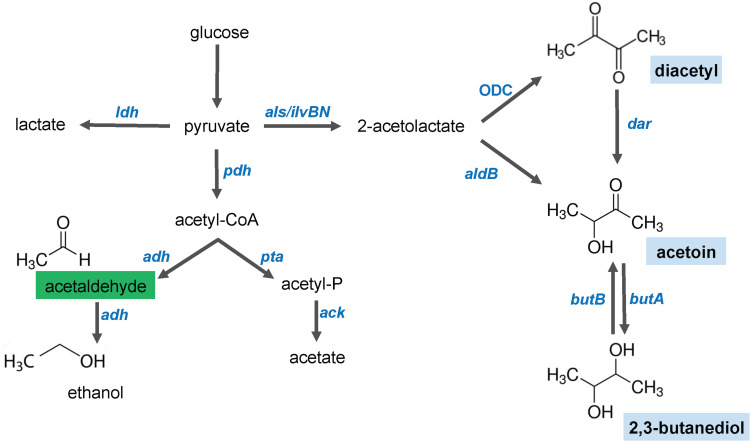
Schematic illustration of the glucose fermentation end products by *Lactococcus lactis*. After glucose is internalized in the cell, its breakdown results in pyruvate. The pyruvate molecules can be converted to several end products. Lactate is the main product of lactate dehydrogenase (*ldh*). Under aerobic conditions, pyruvate is decarboxylaxed by the pyruvate dehydrogenase (*pdh*) complex to produce acetyl-CoA. Acetaldehyde (indicated in a green box), ethanol and acetate are products of the activity of phosphotransacetylase (*pta*), aldehyde/alcohol dehydrogenases (*adh*) and acetate kinase (*ack*), respectively. Under aerobic and acidic conditions, a shift toward the 4-carbon compounds (indicated in blue boxes, diacetyl, acetoin and 2,3-butanediol) occurs. Diacetyl is produced by oxidative decarboxylation (ODC). Acetoin can be produced by activity of a 2-acetolactate dehydrogenase (*aldB*) or by diacetyl reduction by the diacetyl reductase (*dar*). 2,3-butanediol is produced by the acetoin dehydrogenase (*butA*), but this reaction is reversible and 2,3-butanediol can be converted into acetoin by the 2,3-butanediol dehydrogenase (*butB*).

In view of the extreme volatility of acetaldehyde, the dose-dependent fluoresce readouts with this compound are likely to be inaccurate. Therefore, we further characterized only the diacetyl biosensors. Diacetyl is produced as a secondary metabolite during fermentation by some species of the LAB family (Figure 4). The condensation of two pyruvate molecules by the enzyme 2-acetolactate (2-AL) synthase produces 2-AL (Aymes et al., 1999). Then, two alternative paths for conversion of 2-AL to acetoin are possible. The first path is the non-enzymatic oxidative decarboxylation (ODC), which yields diacetyl. Subsequently, diacetyl can be converted to acetoin by a diacetyl-acetoin reductase (Dar) (Rattray et al., 2000). The second path is via the 2-AL decarboxylase (AldB), which yields acetoin. And last, ButA can reduce acetoin to 2,3-butanediol.

**FIGURE 5:**
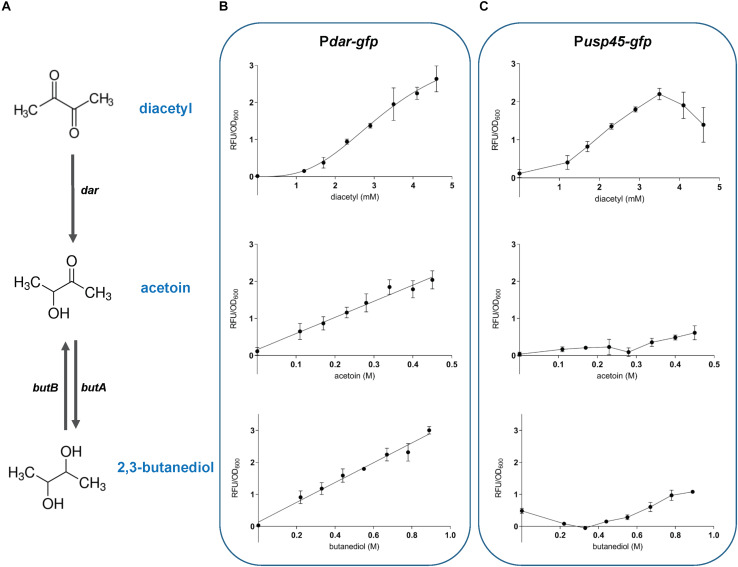
Inducer-dependent orthogonality of the diacetyl-inducible sensors. The response of *L. lactis* sensor strains *Pdar-gfp* and *Pusp45-gfp* to diacetyl, acetoin and 2,3-butanediol **(A)**, diacetyl degradation pathway in *L. lactis*. The enzymes involved in diacetyl degradation include acetoin (diacetyl) reductase (*dar*), acetoin reductase (*butA*) and 2,3-butanediol dehydrogenase (*butB*) **(B,C)**, Dose response curves of the sensor strains, illustrating the correlation between inducer concentration and fluorescence output. Population-level normalized GFP expression (RFU/OD_600_) of bacterial cultures, P*dar-gfp* **(B)**, and P*usp45-gfp* **(C)** strains, in CDM with increasing concentration of diacetyl (1.2 to 4.6 mM; plots at the top), acetoin (0.1 to 0.45 M; plots in the middle), and 2,3 -butanediol (0.2 to 0.9 mM; plots at the bottom). The compounds were added at time zero. Dots represent the average values of independent experiments (*n* = 3). Error bars represent standard deviation (SD) of the mean values of the three independent experiments.

To evaluate the specificity and sensitivity of the diacetyl biosensors, we selected the strains P*dar-gfp* and P*usp45-gfp* based on the clear separation obtained between the treated and untreated samples by flow cytometry analysis. The correlation between inducer concentration and fluorescence output is illustrated in the dose-response curves in Figures 5B,C. The diacetyl curves show that P*dar-gfp* responds to diacetyl in the range of approximately 1.2 to 4.6 mM for a linear fluorescence output. The minimum concentration of diacetyl to activate this biosensor is approximately 1 mM. The dynamic range of P*usp45-gfp* is shorter and thus, its dose-response curve shows its response to the compound in the range of approximately 1.2 to 3.5 mM for a linear fluorescence output. Since *usp45* is a highly expressed gene, we speculate that higher induction levels result in a toxic concentration of GFP.

To evaluate the inducer-dependent orthogonality, we tested the response of our biosensors to acetoin and 2,3-butanediol. As mentioned above, these molecules are degradation products of diacetyl, and may activate the diacetyl-induced promoters. The dose-response curves of P*dar-gfp* show cross-induction by acetoin and 2,3-butanediol, with a remarkably concentration-fluorescence linear response in the range of 0.1 to 0.45 M and 0.2 to 0.9 mM of acetoin and 2,3-butanediol, respectively. This is in contrast to P*usp45-gfp*, which shows no cross-induction to these compounds. We observed that acetoin and 2,3-butanediol that cause the same inhibitory effect on *L. lactis* growth. To discard the possibility that the inhibitory effect on growth affects the GFP expression, the fluorescence readout was evaluated at the concentrations of diacetyl, acetoin and 2,3-butanediol that originate the same inhibitory effect (cultures reached the same final OD_600__;_ see Supplementary Figure 4). The same cross-induction differences between P*dar-gfp* and P*usp45-gfp* are observed when the strains are subjected to identical growth inhibition. These results are in agreement with our previous findings, and since the Dar enzyme is directly involved in the degradation pathway of diacetyl to produce acetoin and 2,3-butanediol (Figure 5A), the cross-reaction of the promoter controlling *dar* expression to these molecules can be explained.

### Diacetyl Producer Strains

We aimed to benchmark the performance of our biosensors to diacetyl produced by wild-type LAB strains. Certain dairy strains, such as *L. lactis* subsp. *lactis* biovar diacetylactis and *Leuconostoc* sp., produce large amounts of 2-AL from citrate metabolism (Laëtitia et al., 2014). Previous studies have reported that LAB produce small quantities of diacetyl and have attempted to increase its production (Boumerdassi et al., 1997; Aymes et al., 1999). Several factors such as pH, temperature, citrate concentration and oxygen are known to affect the diacetyl production yields (Hugenholtz et al., 2000). A total of 10 *L. lactis* subsp. *lactis* biovar diacetylactis strains from different sources were collected (see Table 1). These strains were grown aerobically in M17 medium supplemented with citrate (see section “Materials and Methods”). Initially, we used the classic colorimetric Voges-Proskauer (VP) test to demonstrate acetoin synthesis (Xiao and Xu, 2007; Mcdevitt, 2009). This qualitative method shows that several strains (C17, CRL264, IPLA838, NCDO176+, RR2, and 1816) are able to produce acetoin, and potentially produce diacetyl (Supplementary Figure 5). These results are in agreement with previous studies of the metabolic pathways for flavor formation in these strains. For instance, a previous study of the CRL264 strain identified promoters involved in the biosynthetic pathway of the aroma compounds in this strain (García-Quintáns et al., 2008).

**FIGURE 6:**
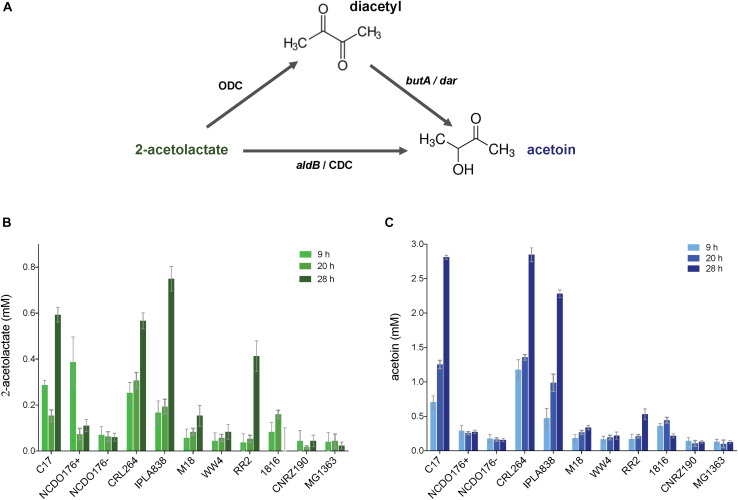
Quantification of pyruvate metabolites (2-acetolactate and acetoin) in *L. lactis* subsp. *lactis* biovar diacetylactis strains. **(A)** 2-acetolactate can be converted into diacetyl and acetoin; diacetyl can be reduced into acetoin. **(B,C)** concentration of 2-acetolactate (plot with bars in green) and acetoin (plot with bars in blue) in bacterial supernatants, respectively. Concentrations of the compounds were obtained at three different incubation times (light color to dark color; 9, 20, and 28 h). The *L. lactis* subsp. *cremoris* MG1363 was included in this analysis as a control strains. Data are presented as mean ± SD. Error bars represent standard deviation (SD) of the mean values of three independent experiments (see section “Materials and Methods”).

To obtain quantitative data of the production of diacetyl, we quantified the pyruvate metabolites 2-acetolactate, diacetyl and acetoin. To this end, we collected and filtered the supernatants of the bacterial cultures at different incubation times (9, 20, and 28 h). The concentrations of the compounds were calculated by the extrapolation method using standard curves (see section “Materials and Methods” and Supplementary Figure 6) (Westerfeld, 1945; Benson et al., 1996). Figure 6 shows that *L. lactis* subsp. *lactis* biovar diacetylactis strains C17, CRL264 and IPLA838 are able to produce high amounts of 2-acetolactate, which might be further converted into acetoin and diacetyl. In contrast to the VP test, by using the quantitative methods we observed that the strains RR2, 1826 and NCDO176 + secrete only small amounts of these compounds. The concentrations of diacetyl in the bacterial samples are below the detection limit (∼0.01 mM).

A previous work on diacetyl overproduction reported that *L. lactis* MR3-T7, a 2-acetolactate decarboxylase mutant with low activity of the lactate dehydrogenase enzyme (LDH), is able to produce up to 6 mM diacetyl (Monnet et al., 2000). Consequently, MR3-T7 was grown as described above, and the diacetyl content in the bacterial supernatant was quantified after 28 h incubation in skim milk and rich medium M17. Diacetyl was present in the M17 supernatant at a concentration of 0.42 mM, whereas the concentration in skim milk was lower than the detection limit. The instability of the LDH activity in this strain that that the authors reported was corroborated by single-colony isolation on LDAH-20-agar plates (see section “Materials and Methods”). A brown color in colonies grown on LDAH-20 indicates low lactate dehydrogenase activity (El Attar et al., 2000). However, this colony selection method has a low reliability and the instability of the LDH activity might explain the differences between the diacetyl concentrations reported for this strain and the diacetyl concentrations obtained in this study.

### Correlation Between Diacetyl Concentration and Biosensor Output

**FIGURE 7:**
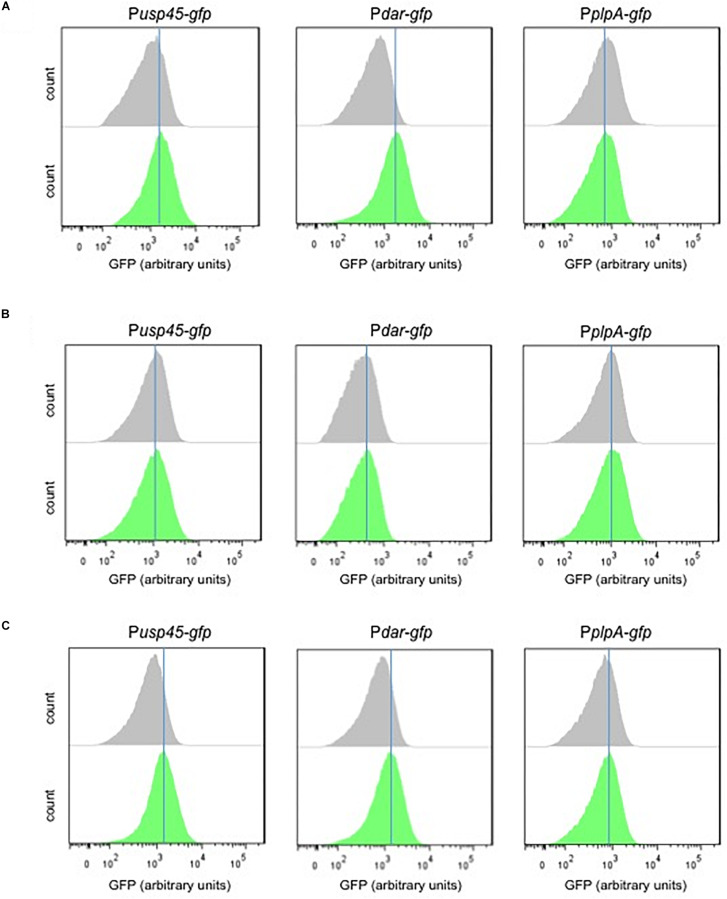
Response of the diacetyl-biosensors to diacetyl-producer supernantants. **(A,B)** Single-cell fluorescence measurements by flow cytometry, in the absence (gray) and presence (green) of supernatant *L. lactis* subsp. *lactis* biovar diacetylactis MR3-T7 grown in M17 or skim milk, respectively. **(C)** Single-cell fluorescence measurements by flow cytometry, in the absence (gray) and presence of diacetyl solution 0.42 mM (green). Note that diacetyl at a concentration 0.42 is used because the M17 supernatant contains diacetyl at the same concentration. Fluorescence measurements were taken at the beginning of stationary growth phase. 10,000 ungated events for each sample are shown.

The supernatants (M17 and skim milk) of the MR3-T7 strain were used to test the diacetyl-induction of the P*dar-gfp* and P*usp45-gfp* biosensors. The biosensors were grown in the presence of the bacterial supernatants (M17 and skim milk) and the GFP expression was measured by flow cytometry. Figure 7 shows a shift in the GFP expression levels of the supernatant (M17)-treated cultures of P*dar-gfp* and P*usp45-gfp*, compared to the P*plpA-gfp* strain. Although minor diacetyl inductions are observed, these increases in GFP expression are not observed when the biosensors are grown with the skim milk supernatants, which showed very low levels of diacetyl. Moreover, we tested the GFP expression of the biosensors by induction with a diacetyl solution as a control (0.42 mM), using the same experimental conditions. Remarkably minor shifts in the GFP expression are also observed in the P*dar-gfp* and P*usp45-gfp*, compared to the P*plpA-gfp* strain. Altogether, these results suggest that the small amounts of diacetyl (0.42 mM) in the M17 bacterial supernatant are responsible for the increase in GFP expression levels by the diacetyl-biosensors P*dar-gfp* and P*usp45-gfp*.

## Discussion

Besides production of lactic acid, LAB produce organic acids, acetaldehyde diacetyl, acetoin, hydrogen peroxide, and bacteriocins (Todorov, 2009). Under certain growth conditions LAB convert pyruvate to several flavor compounds such as acetaldehyde and diacetyl. These flavor compounds are relevant in food industry and its quantification in complex food matrices is a difficult task. On one hand, the classic Voges-Proskauer test is a qualitative method to indicate the presence of acetoin and the potential production of diacetyl in bacterial supernatants. Diacetyl can be converted into acetoin by activity of a diacetyl reductase (Dar). Moreover, a limitation of the VP test is that several factors (experimental conditions such as the incubation time) can affect the results interpretation. On the other hand, the existing quantitative methods require tedious sample preparation protocols and their combination with analytical methods (for instance high-performance liquid chromatography). In this respect, biosensors are molecular tools that can be applied to detect and quantify the presence of metabolites in complex (food) matrices.

Diacetyl is produced as a secondary metabolite during fermentation by some species of the LAB family. While the production of this compound is thought to be a strategy to store energy after carbohydrate depletion (Thomas et al., 1979) or under aerobic conditions (Thomas et al., 1979), diacetyl has previously been shown to be toxic to bacteria. Although its antimicrobial activity may confer competitive advantages over other bacteria to colonize certain habitats (Hugenholtz et al., 2000), this property may explain why only low levels of diacetyl are produced by the cells. Some dairy strains with high diacetyl production have been reported, for instance strains that lack the enzyme ALDC (Kleerebezemab et al., 2000) or strains that have been engineered to combine NADH oxidase (NOX) overexpression and ALDC inactivation (Hugenholtz and Starrenburg, 1992; Curic et al., 1999). Furthermore, other strains have been produced by random selection of *L. lactis* subsp. *lactis* biovar diacetylactis mutants, which showed overproduction of 2-AL and diacetyl (3 mM) due to ALDC deficiency and low lactate dehydrogenase activity (Monnet et al., 2000). To date, the highest titer of diacetyl (95 mM) has been obtained by a combination of engineering strategies and biocompatible chemistry *L. lactis* using bioreactors (Liu et al., 2016).

In this work, we have developed and applied transcriptional biosensors to facilitate the detection of diacetyl and acetaldehyde. The construction of the biosensors is based on the identification of up-regulated transcriptional units by a transcriptome analysis of *L. lactis* exposed to the compounds of interest. Two biosensors consisting in transcriptional fusions (Pr*-gfp*) were obtained and tested for their response to diacetyl (P*dar-gfp* and P*usp45-gfp*) and acetaldehyde (P*butAB-gfp* and P*1772-gfp*). Due to the high volatility of acetaldehyde, we further characterized the diacetyl biosensors for their kinetics (required time to respond to a change in the concentration of the compound), dynamics (range of compound concentration that results in a linear fluorescence readouts) and orthogonality (specificity to the compounds of interest). We evaluated the production of acetoin and diacetyl by *L. lactis* subsp. *lactis* biovar diacetylactis strains. However, only low levels of diacetyl were obtained in standing *L. lactis* cultures under the growth conditions tested in our study. Nonetheless, we benchmarked the performance of the biosensors to respond to a *L. lactis* supernatant with a diacetyl concentration of 0.42 mM. Our results suggest that the biosensors can be applied to quickly detect semi-quantitatively the presence of diacetyl.

The presence of diacetyl is commonly associated with dairy products because it is an important aroma compound found in cheeses, butter, and yogurt. Nonetheless, it is present at variable concentrations in dairy products. For instance, the amounts of diacetyl reported for yogurt range from 200 to 3000 μg/g (2.1–31.4 mM) (Shibamoto, 2014). Likewise, diacetyl is present in starter distillates (SD) at a concentration range from 1.2 to 22,000 μg/g (0.00001–0.22 M). In this study we report that the *L. lactis* P*dar-gfp* strain responds to diacetyl in the range of approximately 1.2 to 4.6 mM. Therefore, a limitation of our biosensors is that they are suitable for foods containing diacetyl at concentrations within the biosensor response range. Recent studies in biosensors engineering demonstrate that biosensors based on transcription factors can be improved in their sensing properties (e.g., sensitivity, specificity, dynamic range) (Mahr and Frunzke, 2016). Further work is required to optimize the functionality of our biosensors for aroma compounds according to a wider concentration range in the target sample.

With regard to diacetyl production using microorganisms, we propose to produce diacetyl by fermentations in bioreactors in order to obtain higher diacetyl yields. Our biosensors are tools with potential application in both development and optimization of LAB strains capable of producing the flavor molecules diacetyl and acetaldehyde.

## Data Availability Statement

All datasets presented in this study are included in the article/Supplementary Material.

## Author Contributions

JH-V and OK conceived the study and wrote the manuscript. JH-V and AS designed the experiments. JH-V carried out the experiments and analyzed the data. AS and OK provided supervision. All authors discussed the results and commented on the manuscript.

## Conflict of Interest

The authors declare that the research was conducted in the absence of any commercial or financial relationships that could be construed as a potential conflict of interest.

## Footnotes

1 https://www.flowjo.com/

2 https://www.graphpad.com/

3 http://www.r-project.org/
