# Supplementary material for: Development of Lactococcus lactis Biosensors for Detection of Diacetyl
Source: Front Microbiol. 2020 May 25;11:1032. doi: 10.3389/fmicb.2020.01032 (PMC7261850; doi:10.3389/fmicb.2020.01032)
Supplement: Supplementary file 2 [file Data_Sheet_2.PDF]

Supplementary Material

1 Supplementary Data

A differential expression analysis of all the genes is provided as Supplementary Data.

2 Supplementary Figures

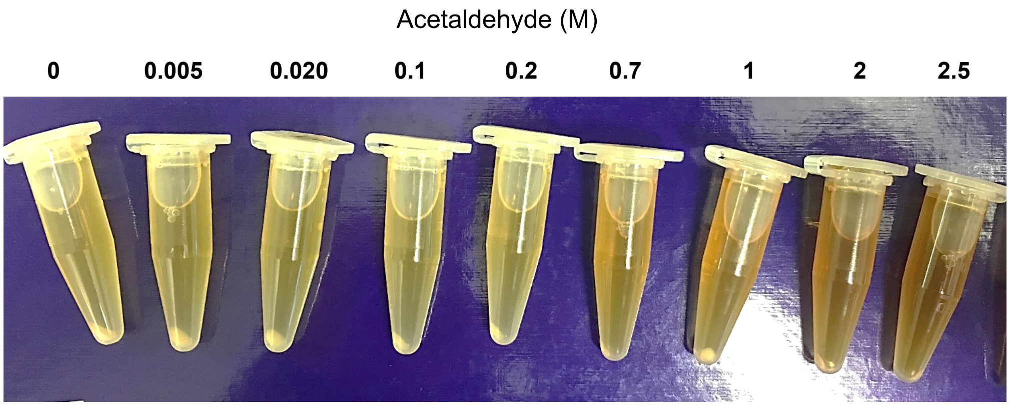

Supplementary Figure 1. **Antibacterial effect of various acetaldehyde concentrations against *L. lactis* cultures.** Due to the high volatility of acetaldehyde, *L. lactis* cells were grown in 1.5 mL Eppendorf tubes tightly closed with parafilm (not shown). Acetaldehyde, M17 medium, tubes and pipette tips were kept at cold temperature to avoid evaporation of the compound.

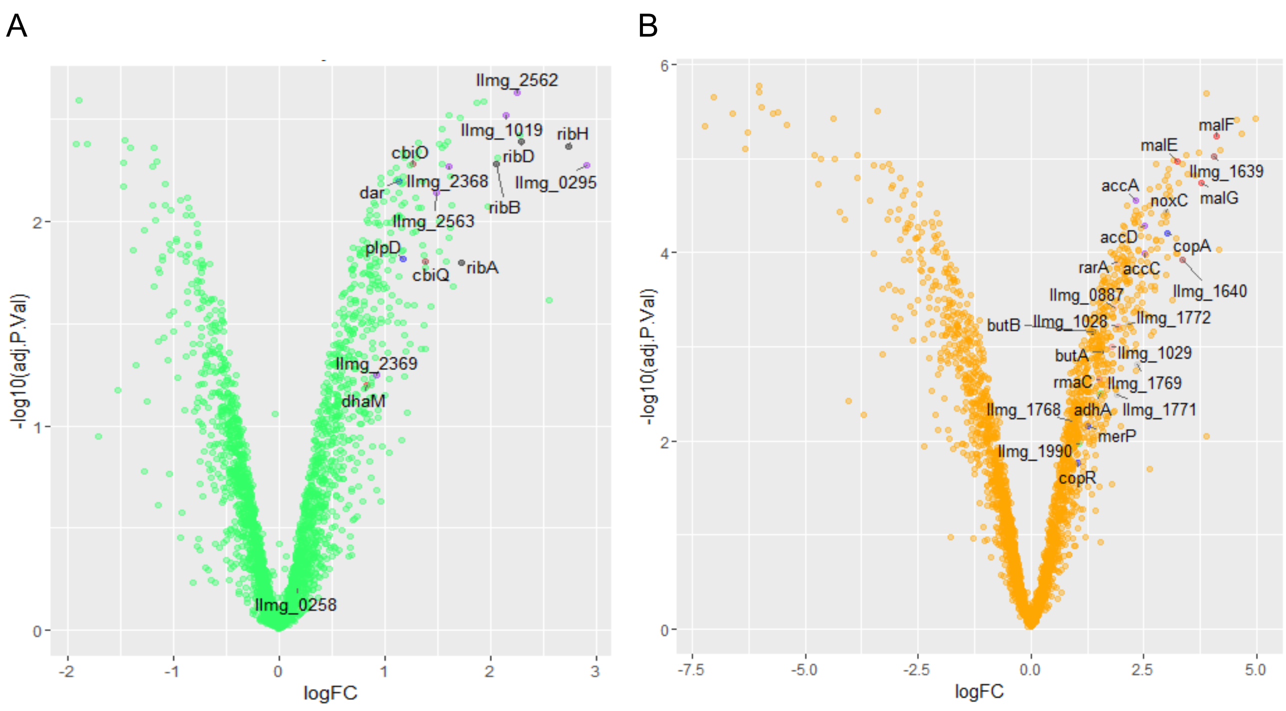

Supplementary Figure 2. Differential expressed genes between compound-treated and untreated *L. lactis* cultures. a and **b,** Volcano plots of differential gene expression of untreated L. lactis cultures versus treated with diacetyl (**a;** green volcano plot) or acetaldehyde (**b;** orange volcano plot). Each point represents the average value of one gene in two replicate experiments. The expression difference is considered significant for a log 2 fold change (x-axis; logFC) ≥ 2.5 and for a P value ≤ 0.05 (y-axis: -log10[adj.P.Val]). Points are colored according to their function category (see Transcriptome analysis file). Names and outlined points represent transcriptional units of one or more genes of interest.

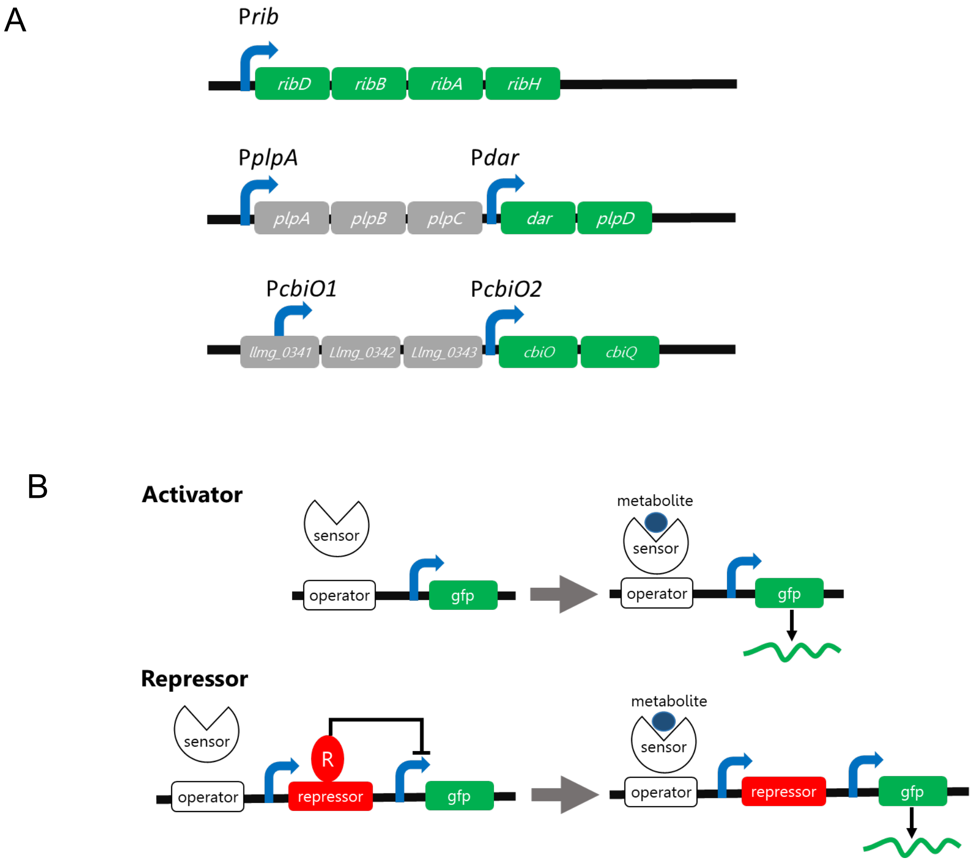

Supplementary Figure 3. **Promoter selection and mechanism of transcription-based sensors. a,** Some up-regulated genes (indicated in green boxes) were predicted to belong to more than one transcriptional units. Then, there are more than one candidate promoters. Therefore, we analyzed two promoters for each of these three transcriptional units to clarify which was the responsive promoter. **b,** The mechanism of transcription-based sensors consists of either activator or repressor. The first scenario involves the direct response of a transcription factor to the presence of the metabolite, which triggers its binding to the operator region in the promoter region and activates *gfp* expression. In the second scenario, the responsive promoter is repressed when the metabolite is not present, but once it is present, the metabolite stimulates the transcription factor to block the repressor expression, and thus, *gfp* is expressed. The diagram of mechanisms is based on a previous work (Mahr and Frunzke, 2016a).

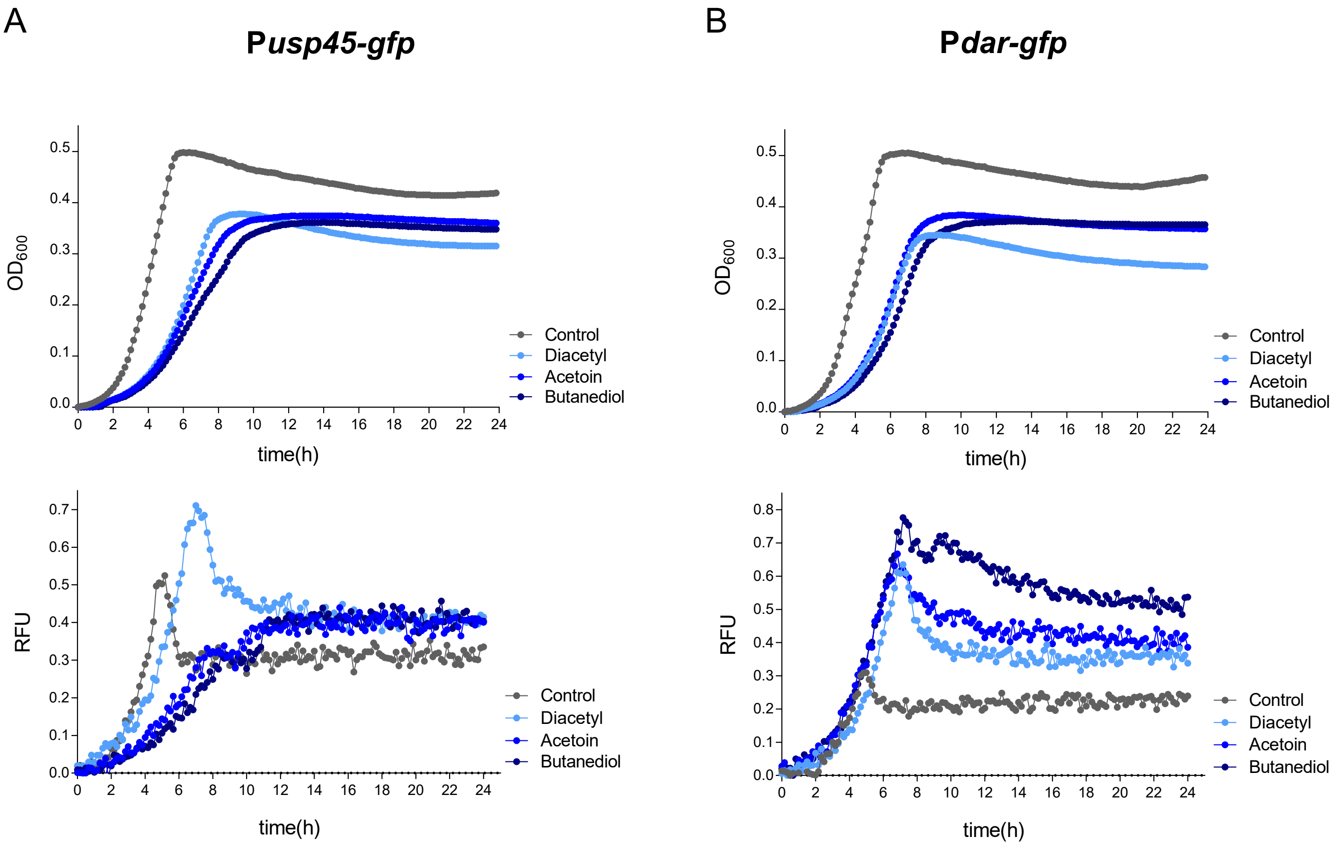

Supplementary Figure 4. **Effect of diacetyl, acetoin and 2,3-butanediol on *L. lactis* MG1363 derivatives growth. a** and **b**, Growth curves (OD_{600}; plots at the top) and fluorescence measurements (RFU; plots at the bottom) of P*usp45-gfp* (**a**) and P*dar-gfp* (**b**) strains. The growth of *L. lactis* in M17 is indicated as control (in grey). A similar growth effect was obtained at different concentrations of acetoin (0.34 mM), diacetyl (3.5 mM) and 2,3-butanediol (0.55 mM).

A

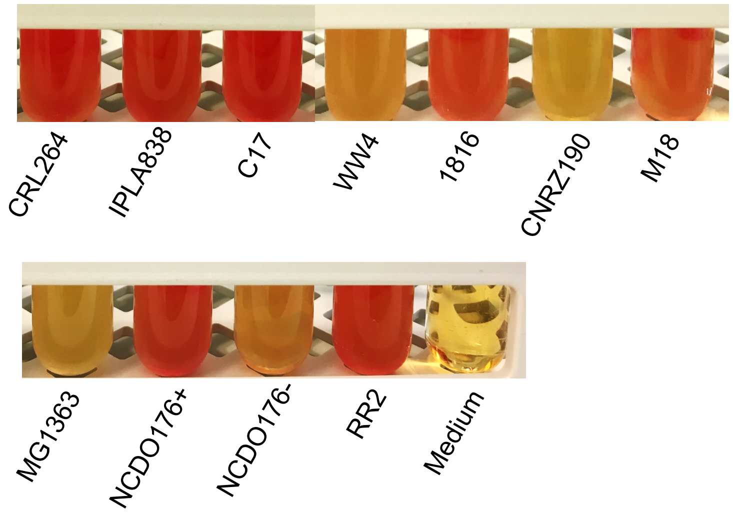

B

Sample	VP
MG1363	-
NCDO176+	++
C17	++
NCDO176-	-
RR2	++
WW4	-
1816	++
CNRZ190	-
CRL264	++
IPLA838	++
RR2	++
M18	+
Medium	-

Supplementary Figure 5. **Voges-Proskauer test.** 2.5 mL of bacterial cultures were used to perform the VP test for acetoin detection, and potential production of diacetyl in *L. lactis* strains. **a,** test tubes with a yellowish color indicates VP negative and tubes with a red color indicates VP positive. **b,** summary of results based on the color observed.

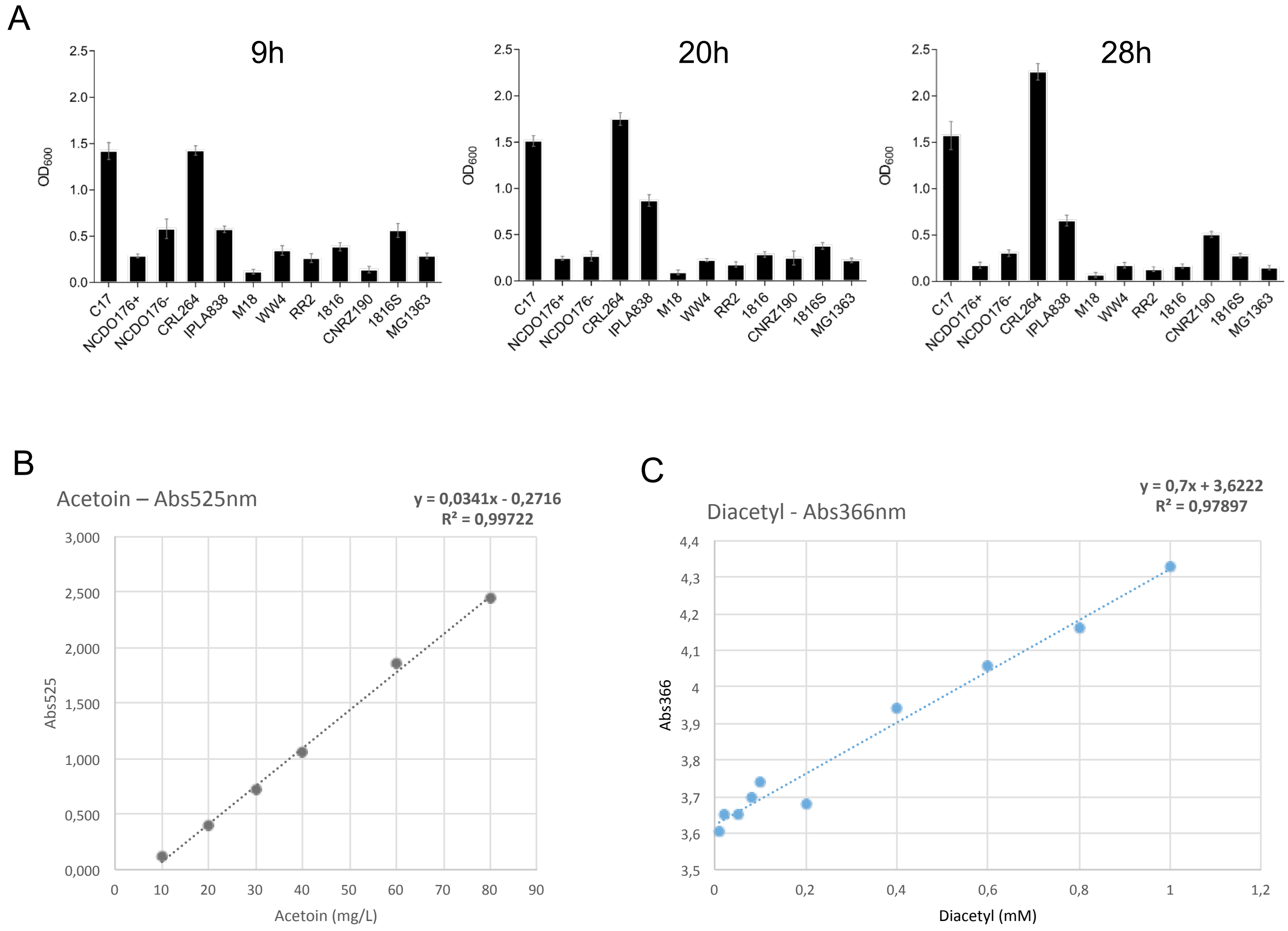

Supplementary Figure 6. **Quantification of pyruvate metabolites. a,** Optical density at 600 nm of the bacterial cultures at each sampling time (9, 20 and 28 h of incubation). Data are presented as mean ± S.D. Error bars represent standard deviation (SD) of the mean values of two independent experiments. **b,** Acetoin quantification standard curve, acetoin concentration (x-axis; mg/mL) and absorbance at 525 nm (y-axis). Linear regression equation is shown (R2=0.997). **c,** Diacetyl quantification standard curve, diacetyl concentration (x-axis; mg/mL) and absorbance at 366 nm (y-axis). Linear regression equation is shown (R2=0.979). Dots represent the mean values. Error bars represent standard deviation (SD) of the mean values of three independent experiments.
